# The Challenges and Opportunities in the Clinical Application of Noncoding RNAs: The Road Map for miRNAs and piRNAs in Cancer Diagnostics and Prognostics

**DOI:** 10.1155/2018/5848046

**Published:** 2018-04-30

**Authors:** Preethi Krishnan, Sambasivarao Damaraju

**Affiliations:** ^1^Department of Laboratory Medicine and Pathology, University of Alberta, Edmonton, Canada T6G 1Z2; ^2^Cross Cancer Institute, Edmonton, Canada T6G 1Z2

## Abstract

Discoveries on nonprotein-coding RNAs have induced a paradigm shift in our overall understanding of gene expression and regulation. We now understand that coding and noncoding RNA machinery work in concert to maintain overall homeostasis. Based on their length, noncoding RNAs are broadly classified into two groups—long (>200 nt) and small noncoding RNAs (<200 nt). These RNAs perform diverse functions—gene regulation, splicing, translation, and posttranscriptional modifications. MicroRNAs (miRNAs) and PIWI-interacting RNAs (piRNAs) are two classes of small noncoding RNAs that are now classified as master regulators of gene expression. They have also demonstrated clinical significance as potential biomarkers and therapeutic targets for several diseases, including cancer. Despite these similarities, both these RNAs are generated through contrasting mechanisms, and one of the aims of this review is to cover the distance travelled since their discovery and compare and contrast the various facets of these RNAs. Although these RNAs show tremendous promise as biomarkers, translating the findings from bench to bedside is often met with roadblocks. The second aim of this review therefore is to highlight some of the challenges that hinder application of miRNA and piRNA as in guiding treatment decisions.

## 1. MicroRNAs

### 1.1. Introduction

For a long time, the field of molecular biology has been governed by the central dogma, which can simply be explained as DNA transcribes to RNA and RNA translates to protein. While this still holds true, recent discoveries have added additional layers to this principle. A group of RNAs termed noncoding RNAs have been found to play a role in regulating transcription and translation. These RNAs were previously not recognized to have significant roles in human health and disease, but we now understand that they are involved in diverse roles ranging from gene regulation to alternative splicing to protein translation. Noncoding RNAs are broadly classified into two groups based on their size: long noncoding RNAs (lncRNAs, https://lncipedia.org/, http://www.noncode.org/), which are generally >200 nucleotides, and small noncoding RNAs (sncRNAs), which are generally <200 nucleotides, and both these groups of RNAs function as regulators of gene expression. sncRNAs include subclasses such as microRNAs (miRNAs, http://www.mirbase.org/), PIWI-interacting RNAs (piRNAs, http://genome.ucsc.edu/), transfer RNAs (tRNAs, http://gtrnadb2009.ucsc.edu/), small nucleolar RNAs (snoRNAs, https://www-snorna.biotoul.fr/), small nuclear RNAs (snRNAs, https://www.ensembl.org/info/genome/genebuild/ncrna.html), and small interfering RNAs (siRNAs, https://www.ncbi.nlm.nih.gov/projects/genome/rnai/). Of these, miRNAs, siRNAs, and piRNAs are classified as regulatory RNAs. Several differences and similarities exist between these three RNAs [1, 2]. For instance, siRNAs regulate gene expression largely by degrading the target mRNA, whereas miRNAs and piRNAs regulate gene expression either by degrading the target mRNA or by inhibiting translation. While siRNAs perform autosilencing (genes that siRNAs target and the origins of the siRNA are from the same gene), miRNAs and piRNAs predominantly perform heterosilencing (targets are different from the genes from which they originate). Likewise, similarities and differences exist in the biogenesis pathway of siRNAs and miRNAs, which are detailed in the review by Bartel [2]. The characteristic feature of these three types of RNAs is their interaction with Argonaute (AGO) proteins to guide target-specific gene regulation. Two classes of AGO proteins exist: AGO and PIWI (P-element-induced Wimpy testis) [3]. While miRNAs and siRNAs interact with the AGO class of proteins, piRNAs are found to interact with the PIWI class of proteins.

### 1.2. Discovery of miRNAs

MicroRNAs are small (~22 nt), noncoding, regulatory RNAs that control gene expression posttranscriptionally by binding to the 3′ UTR of mRNA and promote mRNA degradation or inhibit protein translation [2, 4–6]. miRNAs are the most studied class of sncRNAs and are well explored for their roles in various physiological and pathological conditions.

The first miRNA was discovered by Lee et al. and Wightman et al. in 1993, in the context of studies on the growth pattern of *C*. *elegans* [4, 5]. Growth of *C*. *elegans* depends on heterochronic genes such as *lin-4* that turn on and off during the transition to different developmental stages. Loss-of-function mutation resulted in the absence of adult structures in the worm but reiteration of characteristics specific to early stages; that is, in place of adult structures, the nematode develops early stage structures. By contrast, mutations in another gene termed *lin-14* caused the opposite effect—the worms matured prematurely, suggesting that the two genes imparted opposite effects in the development of *C*. *elegans*. It was later discovered that *lin-4* repressed the activity of *lin-14*; however, the mechanism behind this regulation remained elusive. While the Ambros lab had identified that *lin-4* generated two transcripts, one short (~22 nt) and one long transcript, Ruvkun's lab had found the complementary-binding site for the shorter transcript in the 3′ untranslated region of *lin-14*. They also discovered that *lin-4* binds directly to *lin-14* and suppresses the latter's expression, forming the explanation for the molecular mechanism of small RNAs. These RNAs were initially known as small temporal RNAs as their roles were largely believed to be restricted to the temporal development of nematodes. A few years later, Hamilton and Baulcombe observed a similar silencing mechanism in plants, thus expanding the role of these small RNAs to other organisms [7]. Around the same time, Ambros and his team had discovered that the regulation of *lin-4* was not limited to *lin-14* alone but could regulate another gene called *lin-28*, suggesting possible implications on overall gene regulation [8]. In 2000, Reinhart et al. and Pasquinelli et al. reported the discovery of a second miRNA—let-7—and had made a crucial observation that miRNAs were evolutionarily conserved and not specific to nematodes [9, 10]. This marked the beginning of a surge of reports on miRNAs—discovery, biogenesis, roles, and their significance in different conditions—normal and diseased.

### 1.3. Genomic Location of miRNAs

miRNA genes are found across all chromosomes. They may originate from the intergenic regions or genic regions (both protein coding and nonprotein coding) [2, 11–14]. Further, some miRNAs may be in a distant location from other miRNAs, while some others may be in proximity and may exist as clusters. A cluster, as defined by miRBase (http://www.mirbase.org/), is a group of miRNAs that are located within 10 kb of each other [15]. miRNAs belonging to the same cluster may either be cotranscribed or transcribed independently [16–18].

### 1.4. Biogenesis of miRNAs

The biogenesis of miRNA begins in the nucleus where miRNA genes are transcribed by RNA polymerase II or III into long primary transcripts (pri-miRNAs) that are polyadenylated at the 3′ end and capped at the 5′ end [19–21]. miRNAs arising from intergenic regions are transcribed by polymerase II or III, whereas miRNAs originating from intronic regions are transcribed by polymerase II [22]. Pri-miRNAs contain stem loop structures and are cleaved at the stem of the hairpin structure by a cellular RNase class II endonuclease III enzyme called Drosha along with DGCR8/Pasha into hairpin structures called precursor miRNAs (pre-miRNAs) which are approximately 70–120 nt long [20]. The pre-miRNA harbors a 5′ phosphate and a 2-nucleotide overhang at the 3′ end, characteristic of endonuclease III cleavage. Pre-miRNA is then transported to the cytoplasm with the help of Exportin 5 along with Ran-GTP, which is then processed by the cytoplasmic dsRNase III Dicer into approximately 22 nt miRNA: miRNA duplex with 2 nt overhanging at its 3′ end [2, 11]. The duplex is unwound by helicase, and only one mature strand (~20 nt long) enters the multicomponent complex called RNA-induced silencing complex (RISC), which harbors AGO, and the complementary strand is degraded [2]. Most often, the strand with low internal stability at the 5′ end is retained within RISC [23, 24]. The mature miRNA mediates gene expression by binding to the complementary sequence in the 3′ untranslated region of the target messenger RNA (mRNA). Depending on the complementarity shared, the target mRNAs may be degraded (if the two RNAs are perfectly complementary to each other) or the protein translation may be inhibited (if they share imperfect complementarity) [25]. Excellent schematics of canonical pathways of miRNA biogenesis have been published elsewhere [2, 16].

Alternatively, mirtrons (miRNAs present in the introns) may be processed through splicing mechanisms [26]. In this process, pre-miRNAs are generated by splicing of the introns, which are released as lariat structures. Debranching enzymes act upon these lariat structures for their linearization and to form the short hairpin-like forms. The pre-miRNAs thus formed are transported to the cytoplasm and are cleaved by Dicer to form mature miRNAs. An alternate pathway of miRNA biogenesis is provided in the review by Naqvi et al. [27]. This mechanism was discovered in flies and worms and was later observed in different organisms.

### 1.5. Mechanisms of Action

The interaction between miRNA and target mRNAs predominantly occur at the miRNA seed region (2–8 nt in the 5′ end of the miRNA) and the 3′ UTR of mRNA through sequence complementarity [28]. Several in silico databases such as TargetScan (http://www.targetscan.org/) and miRanda (http://www.microRNA.org/) predict the targets of miRNA based on sequence complementarity. Since these are in silico predictions, two approaches are commonly adopted to gain confidence in the predictions—(i) targets which are predicted in multiple databases are used for functional validation or (ii) targets which are in silico predicted are overlapped with differentially expressed genes identified in tissues. The direct interaction of miRNA with its cognate targets is first confirmed using luciferase assays. Functional validations of the targets are performed using gene overexpression or knockout models in cell lines to observe the effect on the phenotype. miRNA-mRNA interactions include both direct and indirect effects on translation [25]. In the direct effects, initiation of translation or postinitiation of translation is inhibited. While in the former the association of ribosomes with target mRNAs is prevented, the latter includes premature ribosome fall-off, reduced/stalled elongation, or cotranslational protein degradation. Indirect effects of miRNA-mRNA interaction include deadenylation, resulting in degradation or increased turnover. These effects occur in the cytoplasm, predominantly in the processing bodies (P-bodies), which are enriched for factors involved in mRNA degradation. The mRNAs whose protein formation is prevented (by direct or indirect effects) may be sequestered in the P-bodies, which can be used later for translation or can be degraded.

Expression of miRNAs is also regulated at different levels—transcription, Drosha processing, transfer of pre-miRNAs from the nucleus to cytoplasm, and Dicer processing and loading into the RISC complex. Regulation of miRNAs are clearly explained in the reviews by Davis and Hata [29], Winter et al. [16], and Slezak-Prochazka et al. [30]. In addition, miRNAs are affected by the presence of single nucleotide variants and mutations in the miRNA regions or in the binding site of miRNAs leading to improper biogenesis of miRNAs or improper regulation of mRNA targets. Thus, a complex but coordinated association between the regulation of and by miRNAs determines the balance between normal and diseased conditions.

### 1.6. Detection and Quantification of miRNAs

Platforms that have been used for gene expression profiling have also been adopted for small RNAs. However, small RNAs pose several challenges for development of a profiling platform [31–34]—(i) the small size of these RNAs makes it difficult to design a complementary probe or a traditional primer, where, often, the size of a probe/primer is equal to or more than the size of small RNAs; (ii) miRNA isoforms can differ by a single nucleotide and this distinction is difficult to obtain unless the platform is highly sensitive to detect even one nucleotide difference; (iii) the GC content of miRNAs vary greatly, thus making it difficult to standardize the melting temperatures for annealing reactions in a genome-wide study; and (iv) rapid rate of discovery, making it difficult to reuse the data generated on platforms using preprinted probes, based on the existing annotation.

Nevertheless, three main platforms used for profiling small noncoding RNAs include quantitative reverse transcription polymerase chain reaction (qRT-PCR), microarray, and next-generation sequencing (NGS) [35]. Every platform has its own merits and limitations, and a summary of these platforms is provided below.

#### 1.6.1. Quantitative Reverse Transcription Polymerase Chain Reaction (qRT-PCR)

One of the commonly adopted techniques is qRT-PCR that relies on cDNA generated from RNA, followed by real-time PCR. However, due to the small size of miRNAs, conversion of miRNA to cDNA is challenging. Therefore, either poly(A) tail is added or a stem loop primer is added, depending on the method adopted for qRT-PCR. Once cDNAs are generated, PCR reactions are performed. The amplified products are usually detected either by SYBR green or using TaqMan probes. While qRT-PCR offers the advantage of being highly sensitive and specific with a high dynamic range (6 orders of magnitude), this technique has low throughput, and only a limited number of RNAs can be interrogated on this platform [31] due to assay design limitations. It is a labor-intensive method, especially when the experiment includes higher number of samples and the optimal reaction conditions may vary according to sequence-specific differences. The difficulty in designing optimal probes for detecting small RNAs is higher when the miRNAs differ only by one or two nucleotides. Although profiling of multiple miRNAs on a single plate is frequently seen in the literature [36, 37], there is a limitation on the number of miRNAs that can be detected, and this approach may still not cover the entire genome. A major problem with qRT-PCR method is the choice of internal reference used [38]. There is no standard reference gene available yet, and the choice of reference gene thus varies from study to study. One of the common normalizers used is RNU6 [39]. For these reasons, this platform may be best suited to validate or probe for candidate molecules and may be especially useful when the sample amount is limiting.

#### 1.6.2. Microarray

This platform is based on hybridization of probe and target sequences, which are complementary. The technique of printing hundreds to thousands of probes on a chip is perfected and has evolved over the past 15 years. The small size of RNAs poses challenges in this technique as well [34]. Microarrays are less sensitive when compared to qRT-PCR [31, 33], and as with qRT-PCR, the analysis might be restricted to a single class of molecules (e.g., miRNAs alone), in a specific genome build. A platform with low sensitivity will generate many false negative calls, whereas higher sensitivity and reduced specificity would result in a higher number of false positives. The chance of identifying novel RNAs is minimal, and capturing of RNAs with single nucleotide differences is challenging. The dynamic range of this platform is moderate (4 orders of magnitude), but it allows profiling of higher number of miRNAs (compared to qRT-PCR) [31, 40, 41]. The lack of ability to perform absolute quantification of molecules renders it more suitable for comparing relative abundances of molecules that fall within the dynamic range of the platform between two conditions such as normal and diseased. Microarray-based profiling techniques require specialized equipment for hybridization, fluidics stations, incubators, and scanners. The data from high-density arrays also require knowledge of appropriate bioinformatics platforms. Commercially available informatics platforms, for example, the Partek Genomics Suite (http://www.partek.com/pgs), are well designed for use by biologists.

Although both qRT-PCR and microarray have their own advantages, these methods rely on a predetermined set of RNAs based on a specific genome build, thereby leaving us blinded to the functions of other RNAs that cannot be captured because of platform limitations.

#### 1.6.3. Next-Generation Sequencing (NGS)

NGS refers to the sequencing of millions of reads in parallel, yielding higher throughput [40] and more coverage, necessitating the use of powerful computing skills and algorithms for analysis [32]. Several platforms are available for NGS including Illumina (https://www.illumina.com/), Roche (http://sequencing.roche.com/), and Ion Torrent (https://www.thermofisher.com/). These platforms incorporate the sequencing-by-synthesis method that helps in detecting known and unknown RNAs.

The advantage of NGS is that it offers absolute quantification of molecules, higher coverage, and high sensitivity and specificity [35]. It does not require the knowledge of genomic annotation (prerequisite for qRT-PCR and microarray), and the reads can be assembled de novo [42]. This platform overcomes the problems of hybridization encountered in the sequencing technique, is capable of capturing reads with even a single nucleotide difference, and is useful for identifying novel RNAs. It exhibits high dynamic range (>10 orders of magnitude), enabling quantification of low amounts of molecules, and allows parallel quantification of multiple RNA types not restricted to one particular class. Since NGS does not depend on any particular genome build, reanalysis of the existing data based on the current or future annotations of the genome (genome build) is possible. However, sequencing biases may be introduced due to the number of steps involved in sample preparation; data analysis and interpretation is complex due to the large volumes of data being generated. As with all high-throughput techniques, NGS is also not free from batch effects and is discussed later in the review. Of the three profiling platforms, NGS is also the most expensive, but it offsets the costs by allowing mining of all small RNA classes which is not possible on microarray or qRT-PCR platforms.

With several user-friendly bioinformatics platforms now available for data analysis, complexity of data and its mining, once considered a limitation for NGS, has now been overcome [32]. However, different researchers employ different analytical steps starting from aligning the reads to normalization to statistical tests used for identifying differentially expressed RNAs. For instance, several commonly used normalization methods and packages include RPKM [43], DESeq2 [44], edgeR [45, 46], and TMM [47]. There are advantages and disadvantages for each of these, and there is no clear consensus on the best method for NGS data. Similarly, the statistical test employed for identifying differentially expressed RNAs also remains debatable, mainly because of the distribution of reads. While most of the microarray data are log transformed, mainly to bring the data to normal distribution, the use of log transformation is questionable for NGS data. NGS data is count data, and when normalized, it does not fall into normal distribution. Therefore, several methods have to be tried to gain confidence in the results, and regardless of any method that is used, one needs to validate the signatures using a different platform and in different cohorts, preferably using external (geographically distinct regions and diverse population ancestries) validation datasets.

In addition to the three profiling methods mentioned above, other methods are continuously being developed for profiling miRNAs. Some of these methods include NanoString (https://www.nanostring.com/), digital PCR, and multiplex arrays.

### 1.7. miRNAs as Contributors to Tumorigenesis

Since their discovery in 1993, miRNAs have been studied in great depth [48] for their role as key players in normal developmental processes [49], including cell growth and apoptosis [50, 51], hematopoietic cell lineage differentiation [52], muscle cell proliferation and differentiation [53], and also in cancer [54, 55] as well as cardiovascular [56, 57], autoimmune [58, 59], and neurodegenerative diseases [60, 61]. miRNA deregulation in cancer was first demonstrated by Calin et al. [62], wherein the authors observed a deletion at the 13q14 locus in chronic lymphocytic leukemia, which also harbored the miR-15/16 cluster. Reduced expression of miR-15/16 in tumors and their potential to target antiapoptotic target Bcl2 suggested the role of miRNAs as potential tumor suppressors. Conversely, some miRNAs are upregulated in cancers. For example, miR-155 is overexpressed in human B cell lymphoma. Further, miR-155 transgenic mice were observed to favor B cell proliferation, followed by B cell malignancy [63]. Inhibition of miR-21 (a miRNA that is commonly overexpressed in most tumors) led to regression of tumors and overexpression of miR-21 led to the B cell malignant phenotype [64]. These early studies suggested the possible role of miRNAs as tumor suppressors and oncogenes and thus paved the way for a surge of reports confirming these roles. Some miRNAs such as miR-221 have also been found to have dual roles of both oncogenes and tumor suppressors depending on the cancer type in which they are expressed [65–67]. It is now estimated that miRNAs regulate approximately 60% of the protein-coding genes [68, 69]. With an increasing number of studies devoted to miRNAs, the role of miRNAs began to expand, and we now recognize their critical contribution to tumorigenesis—they are important in every hallmark of cancer [70, 71], including evading growth suppressors, avoiding immune destruction, enabling replicative immortality, tumor-promoting inflammation, activating invasion and metastasis, resisting cell death, and sustaining proliferative signaling.

### 1.8. miRNAs as Cancer Biomarkers

A biomarker is defined as “a characteristic that is objectively measured and evaluated as an indicator of normal biological processes, pathogenic processes, or pharmacologic responses to a therapeutic intervention” by the NIH Biomarkers Definitions Working Group [72]. Biomarkers that can aid in disease identification are termed diagnostic biomarkers, whereas biomarkers that guide treatment decisions can be broadly classified as either prognostic or predictive biomarkers. While the former class predicts progression or outcome in an untreated individual, the latter provides information on the likely benefit or detriment from treatment. Therefore, prognostic factors are helpful in identifying patients who are at risk for recurrence and/or death, which may eventually help in modifying treatment modalities, whereas predictive factors may aid in identifying patients who are likely to respond to a treatment. Some of the characteristics of an ideal biomarker include [73] the following: (i) unique expression profile reflecting the diseased condition and altered expression levels in the disease tissue, when compared to the normal tissue, (ii) reliable in identifying the condition, (iii) high stability, and (iv) highly specific and sensitive. miRNAs are highly stable, even in formalin-fixed and paraffin-embedded (FFPE) specimens preserved for over 20 years [74]. Further, miRNAs are stable in body fluids [75] such as plasma [76], serum [75], saliva [77], and urine [78]. They are known for their tissue specificity and can be easily detected by multiple profiling platforms. All these factors make a strong case of why miRNAs are suitable candidates for biomarkers.

#### 1.8.1. miRNAs as Diagnostic Biomarkers

miRNAs are capable of distinguishing between different tumor subtypes. For instance, miRNAs were found to be differentially expressed between the basal and luminal subtypes of breast cancer, and these findings were also validated in an independent data set [79]. miRNAs have been identified as classifiers of histological subtypes of breast cancer. Nine miRNAs (miR-210, let-7d, miR-181a, miR-221, miR-10b, miR-126, miR-218, miR-335-3p, and miR-143) together as a composite signature were found to distinguish ductal carcinoma in situ and invasive ductal carcinoma [80]. Similarly, miRNAs were found to distinguish between different subtypes of renal cell carcinoma (RCC) [81]. Using a decision tree classifier, the identified miRNA signature showed a sensitivity of >95% for distinguishing normal from RCC and papillary RCC and showed 100% sensitivity for clear cell RCC and in distinguishing oncocytoma from chromophobe RCC subtype. Crossvalidation showed 90% accuracy. One of the important roles for miRNA signature is to identify tissues of origin for metastatic cancers of unknown primary site. Ferracin et al. identified a 47-miRNA signature to estimate the tissue of origin probability [82]. This signature identified primary cancers with 100% accuracy and metastatic cancers with 86% accuracy. Independent validation of this signature resulted in 86% accuracy in identifying metastatic cases, and prediction of cancer of unknown primary tumors reached probabilities higher than 90%.

#### 1.8.2. miRNAs as Prognostic Biomarkers

miRNAs have been found to be associated with various clinicopathological factors such as tumor size and stage and as well as metastasis. Research focusing on this aspect of miRNAs has intensified over the years, and now we have mounting evidence associating the expression of miRNAs to clinical outcomes, suggesting their role in cancer prognostication. Let-7 was the first miRNA discovered to have prognostic significance [83]. Expression of let-7 was significantly lower in lung cancer patients, and those patients with low expression of let-7 had shorter survival. Further, let-7 was also shown to be an independent prognostic factor after adjusting for clinicopathological factors. In one of the early studies [84] conducted using microarray profiling, a 13-miRNA signature distinguished (i) chronic lymphocytic patients exhibiting high expression of ZAP-70 (prognostic factor) from patients with low levels of ZAP-70 and (ii) between cases with mutated and unmutated IgV_H_. This signature was associated with disease progression. The observation that the 13-miRNA signature was associated with conventional prognostic factors and was independently associated with disease progression suggested their potential role as independent prognostic factors. In lung cancer, high expression of miR-155 was associated with reduced survival in multivariate analysis [85]. Based on qRT-PCR profiling, Yu et al. identified a five-miRNA signature as an independent prognostic factor for non-small cell lung cancer and also validated this in a validation dataset [86]. A risk score was constructed using the five miRNAs which were significant in univariate Cox analysis. The data was split into low- and high-risk groups based on the median value. Possible prognostic relevance of miRNAs in breast cancer began with the study by Iorio et al. who had identified miRNAs that correlated with breast cancer clinicopathological features, for example, receptor status, tumor stage, vascular invasion, and proliferation index [87]. Similarly, miRNAs discriminated between long-term and short-term survivors of pancreatic cancer [88], showed association with clinicopathological factors of this disease [89]. Likewise, using RT-PCR assays, a seven-miRNA signature associated with overall survival and relapse-free survival of gastric cancer patients [90]. Following some of these early studies and with the intervention of newer and advanced technological platforms to profile miRNAs, several studies reported prognostic markers for various cancer types. Recently, studies have shown the utility of sequencing platforms to identify miRNAs showing clinical relevance. For instance, we have sequenced miRNAs using the Illumina Genome Analyzer platform to identify prognostic markers for breast cancer [39]. Similarly, Roth et al. have identified prognostic miRNAs in the blood of primary lymphoma patients using Illumina HiSeq [91]. Although this is encouraging, we still need to travel a long path in identifying clinically applicable miRNA signatures. Some of the challenges and pitfalls in identifying robust biomarkers are discussed in a later section in this review.

### 1.9. miRNAs in Cancer Therapeutics

Although treatment options for cancer are fairly well developed, there is always a constant need for alternative treatment regimens to further improve survival and quality of life. miRNAs mainly function either as tumor suppressors or as oncogenes. Therefore, the motto for designing therapies using miRNAs would either be to restore tumor suppressive miRNAs using miRNA mimics or to suppress oncogenic miRNAs using inhibitors.

#### 1.9.1. miRNA Mimics

miRNA mimics are chemically synthesized double-stranded miRNA-like molecules designed for gene-silencing purposes [92]. These are generally ~22 nucleotides long and are modified slightly from endogenous miRNAs to enhance stability and loading of miRNA in a RISC complex [93, 94]. One of the ways through which miRNA mimics can be delivered is through the use of nanoparticles. To achieve target specificity, these nanoparticles are coated with antibodies that can recognize tumor-specific antigens, thus binding specifically to tumor cells and delivering the harbored miRNA mimics to tumor cells. One of the well-studied examples is miRNA-34a in neuroblastoma. miR-34a is a proapoptotic tumor-suppressive miRNA that targets multiple genes including MYCN and BCL2 that was encapsulated in a nanoparticle coated with antibodies to disialoganglioside GD2. Delivery of miR-34a through this mode showed multiple pathway regulation such as increased apoptosis and decreased angiogenesis [95]. Intranasal administration of let-7 has also shown to decrease tumor formation in lung cancer-positive mouse models exhibiting mutation for the K-ras oncogene [96]. Expression vector-based gene regulation has also emerged as a means of inducing higher copies of synthetic miRNAs for effective target regulation. Chung et al. demonstrated this using the RNA polymerase II expression vector based on the noncoding RNA BIC [97]. miR-155 precursor sequences are found within BIC, and it was found that the expression of the third exon of BIC is adequate for generating miR-155 copies. Using this premise, a vector capable of expressing two different miRNAs from a single transcript that can effectively inhibit at least two different mRNA targets was constructed. Recently, Esposito et al. identified aptamer-miRNA conjugates as novel tools for *in vivo* targeted delivery of miRNAs with therapeutic benefit [98]. They demonstrated this potential using a tumor suppressor miRNA: let-7g which was conjugated to an aptamer that can selectively bind to tumor cells expressing oncogenic receptor tyrosine kinase Axl. Delivery of this conjugate resulted in marked reduction of tumor cell survival, migration, and growth in a xenograft mouse model of lung cancer. Another approach for delivering miRNA mimics is through the use of liposomes [99]. This was demonstrated in lung cancer models for delivering miR-29b through cationic lipoplexes. miR-29b is a tumor suppressor that is downregulated in cancer. By increasing the expression of miR-29b, lung cancer growth was reduced by about 60%.

#### 1.9.2. miRNA Inhibitors

Inhibitory strategy works mainly based on antisense oligonucleotides (also called as anti-miRs) that include locked nucleic acids (LNA) anti-miRNAs, antagomiRs, miRNA sponges, miRNA masks, and small molecule inhibitors of miRNAs. Anti-miRs work by binding with the mature miRNAs and thereby blocking their interaction with their respective targets. Importantly, anti-miRs possessing 2′-O-methoxyethyl and phosphorothioate modification have been demonstrated to block let-7 activity in *C*. *elegans* [100]. A classic example of LNA is the inhibitory strategy developed against miR-122 for hepatitis C infection. This miRNA is in clinical trials currently and has so far showed positive results, in terms of reducing the viral load in a dose-dependent manner, thus reducing the likelihood of developing hepatocellular carcinoma [101, 102].

Multi-miRNA therapy, where two or more miRNAs act as therapeutic agents, can be used to modulate a mixture of mRNAs and thereby modulate multiple pathways. Similarly, miRNAs can be combined with siRNAs to target a particular gene and boost the inhibition since both these molecules work on similar principles [48]. miRNA sponges are artificially designed multiple complementary target sites for specific miRNA binding, thus preventing the binding of miRNAs to naturally occurring complementary sites in mRNAs. The advantage of this method is that since sponges have multiple miRNA-binding sites, the designed RNAs can sponge the effect of a family of miRNAs sharing the same seed sequence [103]. Naturally occurring RNAs that act as sponge RNAs are the long noncoding RNAs and some transcribed and processed pseudogenes [104, 105].

#### 1.9.3. miRNA-Based Alternative Therapeutic Options

A unique strategy to target miRNAs is through the use of small molecule inhibitors of miRNAs. These agents regulate the transcription or the biogenesis of miRNAs rather than inhibit the interaction of miRNA with its target. A specific example is enoxacin, which by binding to TRBP (the human immunodeficiency virus-transactivating response RNA-binding protein) enhances the production of tumor suppressor miRNAs and inhibits tumor growth in a broad spectrum of cancer types [106]. Likewise, polylysine and trypaflavine inhibit miRNA-mediated gene regulation and thereby attenuate tumorigenic conditions. While the former molecule inhibits Dicer-mediated processing, the latter acts by moderating the association of miRNA with Argonaute proteins [107]. Another strategy to reduce the effect of miRNA on tumor growth or tumor-associated systemic effects is through blocking the production of exosomes or microvesicles and/or blocking the miRNAs harbored within these vesicles. miRNAs act as effective mediators of communication between different cells or tissues. miR-21 and miR-29a released from tumor cells in exosomes can act as ligands that can bind to TLR7 receptors present on the surface of macrophages residing in the tumor microenvironment. This binding triggers an inflammatory reaction that intensifies tumor growth. Therefore, blocking these secreted miRNAs can emerge as a potential therapeutic option [48].

## 2. PIWI-Interacting RNAs (piRNAs)

### 2.1. Introduction

piRNAs are a recently discovered (2006) class of small noncoding regulatory RNAs that are slightly longer (25–32 nt) than the 18–25 nt long miRNAs [108–111]. Similar to miRNAs, piRNAs also interact with the AGO family of RNA-binding proteins to guide target-specific gene regulation, specifically the PIWI subclass of AGO proteins. In contrast to the AGO proteins, which are ubiquitously present in all the tissues, PIWI proteins are predominantly present in the germline cells. In 1997, Lin and Spradling [112] observed an arrest in the self-renewing capacity of germline stem cells present in *Drosophila*, discovering the indispensable role of PIWI proteins in germline maintenance. This observation was confirmed by Cox et al. [113], who also showed that *PIWI* is present both in somatic cells and in germ cells. While *PIWI* expression in somatic cells adjacent to germ cells is required for germline stem cell maintenance, *PIWI* in the germ cells are essential for embryogenesis. Further, they also demonstrated that in humans, *C*. *elegans*, and *Arabidopsis*, *PIWI* codes for proteins necessary for stem cell maintenance, suggesting an evolutionarily conserved role for PIWI proteins.

### 2.2. Discovery of piRNAs

#### 2.2.1. Discovery of piRNAs in Mouse

In 2006, four groups [108–111] independently identified another important class of small RNAs from mouse testes—the PIWI-interacting RNAs (piRNAs). These RNAs were found to be more abundant than any other class of small RNAs, and it was estimated that every spermatid would contain approximately one million piRNAs. Since the mouse-specific PIWI proteins were expressed in a temporal manner during mouse spermatogenesis, it is highly likely that the RNAs interacting with these proteins also show a time-dependent expression. Based on the length, two classes of RNAs were found to be interacting with PIWI proteins: the length of one class of RNAs ranged from 26 to 28 nt and the second class had a size range of 29–32 nt. While the shorter group of RNAs are expressed in the early stages of spermatogenesis (from spermatogonia until the round spermatid appears), the longer class of RNAs appeared later and were more abundant in the adult testes. Several features of this class of RNAs were reported: (i) largely present in germ cells; (ii) expressed in a developmentally regulated manner; (iii) mapping to the genome in clusters; (iv) strong strand bias, showing a preference for Uridine at the 5′ end; (v) show sense and antisense orientation; (vi) map to the intergenic regions, protein-coding regions, and repetitive sequences; (vii) did not show any stem loop structures, as seen in the primary-miRNAs; (viii) had 5′ phosphate group and 3′ OH group; (ix) showed uneven genomic distribution; and (x) showed poor conservation with distant species but were found to be well conserved within closely related genomes such as mouse and rat. Initially, they were believed to be part of repeat-associated siRNAs (rasiRNAs) as they showed regulation of repetitive elements such as transposons in the germline. Dicer-independent biogenesis of piRNAs and their interaction with PIWI proteins distinguished them from both miRNAs and siRNAs and were thus named as PIWI-interacting RNAs or piRNAs.

#### 2.2.2. Discovery of piRNAs in *Drosophila*

There are two subclades of PIWI proteins in *Drosophila*—aubergine and PIWI—which play important roles in germ cell formation and in their development. These proteins were also found to be essential for silencing the transposons. The mechanism through which PIWI proteins promote gene silencing was not known until 2006 when Saito et al. [114] demonstrated that PIWI proteins associated with a set of small RNAs (25–29 nt) in the ovary of *Drosophila*. These small RNAs were distinct from miRNAs and siRNAs and were found to be derived from repetitive sequences. Further, these RNAs were present in both sense and antisense orientation and possessed a 5′ Uridine bias, suggesting their processing by RNase III enzyme. Immunoprecipitation and northern blot analyses revealed that PIWI proteins did not partner with miRNAs or siRNAs and their association with rasiRNAs was specific. An interesting observation was that PIWI proteins exhibited slicer activity in the nucleus of the cell, in contrast to the AGO protein which exhibited slicer activity in the cell's cytoplasm. In all, Saito et al. demonstrated that PIWI proteins perform gene-silencing activity in the nucleus through their specific association with rasiRNAs. These rasiRNAs, associated with PIWI proteins, were later called as piRNAs.

Brennecke et al. [115] performed a detailed study to identify and understand the small RNAs associated with PIWI proteins. They purified the PIWI protein complexes obtained from the ovary and sequenced the small RNAs found within the complex. Several observations were made: (i) the length of the small RNAs ranged between 23 and 29 nt, (ii) these RNAs showed strong preference for Uridine at the 5′ end, and (iii) most of the sequences could be aligned to annotated transposons or their remnants. An important finding was the identification of genomic origin of piRNAs. piRNAs were found to be present as clusters in discrete loci in the genome, and these were observed to be bidirectionally transcribed. These clusters were often found in the regions of high transposonal activity such as in the pericentromeric and telomeric heterochromatin and in the euchromatin regions [115].

#### 2.2.3. Discovery of piRNAs in Humans

Immunoprecipitation and northern blot analyses revealed the presence of piRNAs in fetal and adult human ovaries and testes [116]. The identified piRNAs exhibited all the characteristics found in piRNAs identified from mice. While more than half of the piRNAs were found to arise from unidirectional-promoter-containing genes, the remaining piRNAs were found to arise from bidirectional-promoter-containing genes. The piRNAs were widely distributed across the autosomes and sex chromosomes. piRNAs were found in higher amounts in adult testes and ovaries and fetal ovaries, when compared to fetal testes. Sexually dimorphic patterns of piRNAs were observed in terms of length, distribution, and expression. In general, piRNAs appeared to be slightly longer in adult testes, when compared to the RNAs obtained from the ovaries. In contrast to what was observed in *Drosophila* or mice, piRNAs in humans mapped to the intergenic regions predominantly and only 2% of the identified piRNAs mapped to the transposable elements. This suggests that the primary function of piRNAs in humans may not include regulating transposons. Ha et al. profiled the piRNAs from human adult testis [117]. All the features of piRNAs reported in humans and other model organisms were confirmed in their study. In addition, genic regions of protein-coding genes were reported as the possible origin and genomic location of piRNAs in humans. Genic regions include 3′ and 5′ untranslated regions (UTRs) and the coding regions. piRNAs were found to be significantly enriched in the 3′ UTRs. However, the expression of these piRNAs did not correlate strongly with the expression of its corresponding host genes, from where the piRNA originated, indicating an alternative mechanism for piRNA generation from protein-coding genes. piRNA transcripts were also observed to be generated from long noncoding RNAs which are >200 nt in length. In addition, we [118, 119] and others [120–123] have suggested that transfer RNAs and small nucleolar RNAs may also be the sources of piRNAs as we observed some of the piRNA genes to exist within the boundaries of transfer RNAs and small nucleolar RNAs [118–123]. However, the process through which piRNA transcripts are generated remains elusive.

### 2.3. Biogenesis of piRNAs

The main difference between piRNAs and miRNAs biogenesis is that piRNAs do not require a double-stranded precursor molecule nor do they require the Dicer molecule for their biogenesis.

The biogenesis pathway of piRNA remains elusive, and the majority of our understanding concerns the *Drosophila* pathway. Often, piRNA biogenesis is also associated with silencing of target genes and requires all three PIWI proteins—PIWI, AUB, and AGO3. While PIWI proteins are found predominantly in the nuclei of germ cells and the somatic cells [124], AUB and AGO3 are found in the cytoplasmic regions of the germ cells [115]. In general, PIWI and AUB show preference for sequences with a 5′ Uridine bias mapping antisense to transposons, whereas AGO3 prefers sequences with adenine at the 10th position, mapping sense to transposons [115]. piRNAs take two routes for their processing: primary synthesis pathway and the secondary pathway/ping-pong amplification [125–128]. It is believed that the primary biogenesis pathway is necessary to initiate PIWI pathways, while the secondary pathway is necessary for maintaining the piRNA levels as well as for target silencing. While we only observe the primary pathway in somatic cells, we observe both primary and secondary pathways in germ cells [129].

Primary synthesis begins with the transcription from piRNA clusters by RNA polymerase II. The exact machinery involved in the processing is not known. However, it has been proposed that Zucchini, an endonuclease enzyme, cleaves the long single-stranded transcript, generating a 5′ phosphate residue. These piRNA-like molecules thus generated are loaded onto PIWI proteins (most often, PIWI and AUB proteins). The 3′ ends, which generally possess extra bases, are trimmed upon their association with PIWI proteins, and the length of a mature piRNA is established at this stage. Factors contributing to 3′ end trimming are not known yet. However, this is followed by 2′-O-methylation by Hen1 to confer stability to the piRNA and generate mature piRNA.

The secondary mechanism, known as the amplification cycle, involves only AUB and AGO3 and not PIWI proteins. piRNAs generated from the primary pathway may enter into the secondary pathway and subsequently bind with Aub. Using piRNA as a guide, the piRNA-AUB pair recognizes a complementary sequence from the transposons. AUB cleaves the complementary sequence, 10 nucleotides from the 5′ end of the primary piRNA. This generates a new piRNA with the 5′ end, which then binds with AGO3, and the same cycle of pairing with target sequencing, trimming, and generation of piRNA continues. In this cycle, the binding of piRNAs to AUB and AGO3 alternate with each other and the sequences that bind to these proteins are complementary to each other. This secondary cycle which serves the dual purpose of generating piRNAs and silencing transposons is thus called as amplification cycle or ping-pong cycle.

A similar biogenesis pathway has been observed in mice. Although there are striking differences in the manner in which piRNAs are generated in *C*. *elegans*, many of the factors identified in flies and mouse have also been observed in *C*. *elegans* (reviewed in [130]). Although there are no conclusive reports on the biogenesis pathway of piRNAs in humans, there are clues to suggest that it may be similar to the process observed in *Drosophila* or mouse [116, 131].

### 2.4. Functions of PIWI Proteins and piRNAs

Functions of piRNAs and PIWI proteins can be classified under two categories—developmental and regulatory (reviewed in [125]). The development functions can be further categorized into germline and somatic functions.

#### 2.4.1. Contribution to Developmental Processes

Our understanding on the development functions of PIWI proteins originates from *Drosophila*, mice, *C*. *elegans*, and other model organisms. The major roles of PIWI proteins in the germline function include the formation of germ cell [132–134], maintenance of germline stem cells [112], meiosis [135], spermiogenesis [136], and oogenesis [112, 137]. Gene knockout and knockin experiments demonstrate the contribution of PIWI proteins in these functions. The significance of PIWI proteins has expanded beyond germ cells to somatic tissues. For instance, they are known to mediate epigenetic regulation and stem cell maintenance in *Drosophila* as well as maintenance of neoblast cells in planaria [138, 139]. The development of ciliates involves germline micronuclei and somatic macronuclei [140]. A certain amount of DNA sequences found in the somatic macronucleus has to be eliminated during sexual reproduction, and PIWI proteins are known to play a major role in DNA elimination.

#### 2.4.2. Contribution to Gene Regulation

PIWI proteins may serve as epigenetic suppressors or activators [141–143], depending on the recruitment of specific proteins. It has also been observed that transposon-coding genes are not methylated in the absence of PIWI proteins, reflecting to a loss of epigenetic control (reviewed in [125]). piRNAs and PIWI proteins may both serve as upstream mediators of epigenetic control and may also be involved in transcriptional gene silencing. The role of piRNAs and PIWI proteins in silencing transposonal activities is well studied. It is believed that piRNAs occur as clusters. A specific example is the flamenco region in flies, which harbors one of the largest piRNA clusters [115, 144]. A disruption in the flamenco region interrupts the production of piRNAs, with a simultaneous increase in transposon activity. Also, the biogenesis pathway of piRNAs also serves a dual purpose—to generate piRNAs and to mediate gene silencing. Cofractionation of PIWI proteins and piRNAs with polysomes has hinted at the possibility of a potential role for PIWI proteins and piRNAs in translational control [110, 145]. One of the important observations is the role of piRNAs in posttranscriptional gene silencing. Although the mechanism still remains unclear, it is believed that piRNAs may act in a manner similar to that of miRNAs. A study from Esposito et al. [146] showed that piR_015520 negatively regulates its host gene, melatonin receptor 1A (*MTNR1A*), offering new functions for piRNAs, similar to miRNAs. Other studies have also confirmed the relationship between piRNAs and its corresponding target mRNAs [147–150], even though it is not known if the piRNAs have any seed sequence that determines its complementary binding with the target mRNA.

### 2.5. Detection and Quantification of piRNAs

Traditionally, piRNAs bound to PIWI proteins were identified by coimmunoprecipitation, followed by sequencing or RT-PCR of RNAs. The piRNAs thus identified were further validated using northern blot analyses and in situ hybridization [108–111, 151]. Similar to miRNAs, piRNAs can also be detected using microarray, sequencing [152], and qRT-PCR. One of the challenges with sequencing piRNAs lies in the binding of adapters to the 3′ end as these RNAs are characterized by the presence of 2′-O-methylation [153]. Therefore, traditional sequencing often leads to underrepresentation of piRNAs. One of the ways to overcome this challenge is through the inclusion of treatment with periodate before adding adaptors during library preparation [153]. This is often followed by confirmation with piRNA databases and/or coimmunoprecipitation with PIWI proteins.

Recently, piRNAs have also been profiled using microarray platforms. The most commonly used microarray platform is the Arraystar platform (Arraystar, Rockville, MD) that has been designed to profile more than 23,000 piRNAs in humans [154–156]. Often studies conducted using microarrays first identify piRNAs of interest and then perform crossplatform validation using qRT-PCR. To date, a qPCR array for piRNAs has not been developed, and investigators need to put in efforts to design individual assays. So far, candidate piRNA molecules have been validated by qRT-PCR using either the SYBR Green method or the TaqMan method using stem loop primers.

### 2.6. PIWI Proteins and piRNAs as Contributors to Tumorigenesis

The contribution of PIWI-piRNA to cancer comes in different layers. First, PIWI-piRNA complexes maintain genomic integrity by silencing transposons through DNA methylation. Transposons are mobile elements that can get transferred and interspersed between different genomic locations, generating insertions, deletions, and other chromosomal aberrations. All of these aberrations are critical for tumor development. Second, the role of PIWI-piRNA in conferring epigenetic modification has been demonstrated in model organisms. PIWI is guided by the piRNA to the genomic region where the piRNA shares complementarity. This interaction helps in recruiting factors necessary for imparting methylation and/or acetylation, which are essential for transcriptional regulation of gene expression [157]. One example of this mode of gene regulation is the silencing of killer immunoglobulin-like receptors (*KIRs*) [158]. Antisense transcripts of *KIR* are processed to piRNA-like molecules. The piRNA-PIWI complex binds to the promoter region and favors recruitment of methylation factors, enabling transcriptional silencing of the gene.

While the role of piRNAs in regulating transposons and in mediating epigenetic regulation is well documented [159], the role of somatic piRNAs in cancer biology is increasingly being recognized [160]. Our knowledge on the significance of piRNAs in cancer originated from delineating their role in germ cell cancers [161]. Although when compared to germline piRNAs, the number of piRNAs are lesser in somatic cells (both level of expression and number of distinct piRNAs), their contribution to tumorigenesis is significant. While germline piRNAs are known for their regulation of transposons, recent studies suggest their role as regulators of gene expression in the somatic cells [148, 162]. Similar to miRNAs, piRNAs, along with their cognate partner (PIWI), have been shown to bind to complementary target sequences and promote mRNA deadenylation or degradation. Similar to PIWI, piRNAs are dysregulated in cancers, when compared to normal tissues. Further, the expression patterns of piRNAs have also been reported to vary across different tissue types, suggesting a possible tissue-specific expression of piRNAs [163]. Likewise, similar to PIWI, recent studies have shown that up- or downregulation of piRNAs may induce imbalance in the cell machinery, causing increased cell proliferation [164], invasion and metastasis [165], decreased apoptosis [166], and so on. With these diverse functions, piRNAs are now classified as “master regulators of gene expression.”

### 2.7. piRNAs as Diagnostic and Prognostic Markers of Cancer

piRNAs are slowly garnering attention as diagnostic and prognostic biomarkers for various cancer types. For example, Cui et al. observed significantly lower levels of piR-651 and piR-823 in the peripheral blood of gastric cancer patients and their association with clinicopathological features [167]. They also obtained an area under the curve value of 0.841, 0.812, and 0.860 for piR-651, piR-823, and piR-651, respectively, combined with piR-823, indicating the diagnostic potential of these piRNAs. Two studies demonstrated association of piRNAs with renal cell carcinoma. In the study conducted by Busch et al., three piRNAs (piR-30924, piR-38756, and piR-57125) were associated with tumor recurrence and overall survival. Two of these (piR-30924 and piR-57125) also emerged as independent prognostic markers [154]. In another study conducted by Li et al., high expression of three piRNAs (piR-32051, piR-39894, and piR-43607) was associated with metastasis, clinical stage, and survival in renal cell carcinoma [168]. In gastric cancer, one piRNA (FR222326) and three piRNAs (FR290353, FR064000, and FR387750/FR157678) associated with overall survival and recurrence free survival, respectively [169]. Similarly, in breast cancer, four piRNAs (hsa_piR_009051, hsa_piR_021032, hsa_piR_015249, and hsa_piR_020541) associated with overall survival and six piRNAs (hsa_piR_017061, hsa_piR_009051, hsa_piR_021032, hsa_piR_004153, hsa_piR_017716, and hsa_piR_019914) showed association with recurrence-free survival [162]. Analogous to miRNAs, piRNAs are stable in biofluids such as plasma and blood [170]. piRNAs isolated from these biofluids were found to be deregulated in patients with colorectal cancer, prostate cancer, and pancreatic cancer [171]. More studies confirming the prognostic potential of circulating piRNAs may add a new dimension to the existing roles of piRNAs.

### 2.8. PIWI-piRNAs and Cancer Therapeutics

It is becoming evident that PIWI proteins and piRNAs are integral components of tumor development. These molecules operate at various levels including transcriptional, posttranscriptional, and epigenetic. Altered expressions of both these molecules have proved to alter cell growth, bringing about increased cell proliferation, decreased apoptosis, and increased invasiveness and motility, all leading to transformation of a normal cell to a malignant cell. Similar to miRNAs, silencing of piRNAs and PIWI may reverse these phenotypes. Dysregulation of PIWI proteins also contribute to altered levels of piRNAs in a cell. Therefore, regulating the expression of PIWI would serve dual purposes—control the expression of piRNAs which otherwise may be dysregulated in a cancer cell and in restoring a cell's balance. Regulation of mRNAs by piRNAs is currently being addressed. While the exact mechanism remains elusive, piRNAs are most likely to interact with mRNAs through complementary sequences and bring about mRNA degradation or translation inhibition, similar to miRNA-mediated regulation. Although not well studied, piRNAs and PIWI tend to share a feedback regulatory mechanism. While the control of PIWI over the expression of piRNAs is quite well known, possible regulation of PIWI expression by piRNAs, in a manner similar to miRNA-mediated regulation, has only been proposed. We had, in our earlier study, observed that (i) hsa_piR_021032 shares a complementary sequence with PIWIL2 and (ii) the expressions of both these molecules were in opposite directions [162]. These observations suggest that piRNAs may directly regulate the expression of PIWI, a mechanism that has not yet been experimentally validated. So far, we do not have any therapeutics developed using these molecules. Silencing or inducing the expression of PIWI/piRNA can alter the recruitment of epigenetic factors and can modify the overall expression levels of PIWI/piRNAs and mRNAs via transcription or posttranscription, thereby influencing the overall development and progression of cancer. The field of piRNAs is relatively young but promising. However, all these observations and evidence highlight the critical role that these molecules play in overall tumorigenesis and emphasize the need to comprehensively understand the mechanisms of these molecules and explore their therapeutic potential.

## 3. Challenges Involved in Biomarker Development

miRNAs belong to a well-studied class of small noncoding RNAs. The role of miRNAs as biomarkers has also been well explored, and several investigators are focusing on comprehensive understanding and utility of these RNAs in clinics. Other classes of small RNAs such as piRNAs have also shown promise as biomarkers, and extensive studies are needed in this direction to translate this finding to clinics. However, many challenges exist and thus hinder the immediate application of these molecules in clinics. Some of these challenges are outlined below.

### 3.1. Choice of Normal Samples

The choice of normal samples chosen for comparison with tumor tissues remains debatable. One school of thought encourages the use of tumor-adjacent normal tissues, as these are scored also for the presence of tumor cells [172, 173]. Since these tissues do not show the presence of tumor cells, these are considered to be a safe choice for comparison. However, another school of thought dismisses the idea of using adjacent normal tissues for two reasons—(a) lack of molecular differences between tumor and adjacent normal tissues against the presence of difference between tumor and normal tissues obtained from apparently healthy individuals [174, 175] and (b) lack of consensus on the distance from the tumor that a tissue can be designated as normal [174, 176, 177]. Studies have been conducted to understand the molecular differences between tumor-adjacent normal tissues, normal tissues obtained from healthy individuals, and tumor tissues. For instance, in a study conducted by Sanz-Pamplona et al. [174], mRNA levels from 98-paired adjacent normal mucosa were compared with colorectal cancer tissues and with 50 colon mucosa from healthy donors. It was observed that a number of genes were activated in the tumor-adjacent mucosa, similar to the tumor tissues, and the activation of these genes was not observed in the normal tissues from healthy donors. In fact, principal component analysis of the three tissue sets identified different clusters for the three tissue types, suggesting that considerable differences existed between the tumor-adjacent “normal” tissues and healthy normal tissues. Further, a total of 895 genes were found to be differentially expressed between the two normal tissues. Functional enrichment analysis and network analysis of the activated genes from adjacent normal tissues identified pathways significant for tumorigenesis, implying that the adjacent normal tissues may mimic the effects of tumorigenesis. The same was validated in publicly available datasets, consistent with the view that adjacent stromal cells crosstalk with tumor cells within this microenvironment (called as “the field effect”). Therefore, use of adjacent normal tissues as a reference may result in misleading interpretations. This bystander effect of tumors on adjacent normal tissues has been observed in other cancer types as well, such as prostate cancer [175, 178, 179] and breast cancer. Similar anomalous patterns of expression were reported even for noncoding RNAs when tumor-adjacent normal versus reduction mammoplasty normal tissues were compared [180]. In support of this general premise, differential miRNA expressions were reported between reduction mammoplasty and tumor-adjacent normal tissues. Differentially expressed long noncoding RNAs were absent when tumor-adjacent normal tissues versus tumor tissues were compared [181]. However, differentially expressed transcripts were identified when tumor tissues were compared with reduction mammoplasty specimens. It is safe to infer from the above examples that where possible, use of normal tissues obtained from apparently healthy individuals may be more appropriate tissue for comparison with tumor tissues [181].

### 3.2. Profiling Platform

miRNAs can be profiled using different platforms including microarray, qRT-PCR, and NGS. Each platform possesses its own advantages and disadvantages, and therefore, the choice of profiling platform is critical for discovery and validation studies. Going forward, while NGS may be the preferred choice for profiling small noncoding RNAs, it may not be easily applicable in the clinics, and therefore, one needs to optimize other platforms such as NanoString to validate the expression of miRNAs obtained from NGS and their applicability to clinic.

### 3.3. Sample Size and Study Design

The number of samples chosen in a discovery cohort is crucial. A biomarker study should represent the population. The higher the sample size, the higher is the statistical power of that study. For instance, identifying differentially expressed RNAs from five samples in each group may give rise to false positive associations and may lead to dimensionality problem, where the numbers of markers are much higher than the number of samples used. Therefore, multiple rounds of validation of signatures in different populations are required to gain confidence in the identified signature.

### 3.4. Study Design and Analysis Methods

Different factors need to be considered when designing a biomarker study. Many studies have used the case-control approach [182, 183]. It is common practice to identify differentially expressed RNAs between the two groups and test the prognostic relevance of differentially expressed RNAs. While it is important that a biomarker shows distinct expression patterns between normal and tumor tissues, it is not necessary that only differentially expressed RNAs will show prognostic relevance. With this objective, biomarker studies have also been conducted using only the tumor tissues and without focusing on the differentially expressed RNAs—a case-only study [86, 90]. To understand the differences and similarities between these two methods, recent studies from our group compared the results obtained from both the approaches and observed that the case-only study is an unbiased approach that identifies more numbers of clinically significant RNAs [39, 118, 119, 162]. The case-only approach not only identifies all the RNAs identified in the case-control approach but also identifies newer molecules that were not differentially expressed.

### 3.5. Batch Effects

Batch effects should be corrected for. Analyzing the data in the presence of batch effects may lead to spurious associations. While it may not be possible to contain for all the source of variations, it is more appropriate to acknowledge, detect, and contain as many sources of technical variations as possible. One of the ways to detect for batch effects would be using principal component analysis, and one of the ways to quantify and remove batch effects would be using ANOVA. Leek et al. clearly explain the causes, effects of, and methods to detect and correct for nonbiological sources of variation [184].

### 3.6. Expression of RNAs

Handling of high-throughput data, especially NGS data, is challenging. A large number of RNAs may be expressed only in one or two samples and that too with very low expression, with one read count. This single count could be due to technical artifacts. Therefore, it may be a good idea to remove the RNAs which have low expression. Some investigators consider the total read count in the dataset, whereas some others consider per-sample read count [185–189]. Based on the objective of the study, one has to determine the cut-off for read counts. For instance, one of the characteristics of biomarkers is that a biomarker should be expressed in most of the samples. In this case, choosing a molecule that is expressed in >90% of the samples may be appropriate [39, 118, 119, 162].

### 3.7. Data Normalization

The choice of normalization method is vital. Several methods exist for normalizing the data generated from microarray as well as from NGS. While most of the microarray studies now employ quantile normalization, the most appropriate method for normalizing NGS data remains elusive. Several choices include RPKM, and TMM. Each of these methods and/or packages has been used by researchers to successfully identify RNAs. However, we do not yet have consensus on which method is more suitable to handle NGS data.

### 3.8. Single versus Panel Markers

The choice of single markers versus combination markers is important. Biology is driven by multiple interacting modules, and the increasing use of panels of mRNA markers for prognosis is recognized; use of a single marker (for instance, miRNA, which has the intrinsic property of pleiotropy) may or may not capture the higher level of complexity inherent to biology. A single miRNA may regulate multiple mRNAs, and multiple miRNAs may target a single mRNA. While some argue the use of single markers as more effective markers, a combined signature of various RNAs such as miRNAs, piRNAs, and mRNAs may more closely reflect the overall biology of the underlying disease.

The confidence in a biomarker study largely depends on its reproducibility in external datasets with large sample sizes. This adds strength to the initial study findings and may eventually lead to generalizing the initial study findings. However, it is not an easy task to obtain an external dataset because several factors need to be considered. These include (a) number of samples available, (b) histological and molecular subtypes of the cancer studied, (c) profiling platform used for the study, (d) number of events (dead/alive, recurrence), (e) the amount of clinical information available and attempts to adjust for confounding factors including sex, (f) number of years of follow-up, and (g) the population ancestry; these may all influence the replicability of findings across laboratories.

## 4. Conclusion

Despite progress in the miRNA field and utility of miRNAs as diagnostic, prognostic, or predictive markers, their adaptation to the clinic is slow, and that is to be expected. More than 100 clinical trials (https://clinicaltrials.gov/) are currently underway for use of miRNAs in cancer and other diseases. Compared to miRNA, the piRNA field is in its infancy, and hence, applications to the clinic will not be seen immediately. The identified and characterized miRNA markers need several independent validations with large sample sizes. Biomarker acceptance will be high if applicable to all ethnic groups. These biomarkers should also undergo extensive validations in prospective cohorts and with clear benefit in terms of positive predictive value and cost benefit over the existing markers currently in use. Last but not the least, change in clinical practice guidelines meets with considerable resistance as this requires extensive education and expertise to accept new diagnostic or prognostic tools across physicians, administrators, and laboratory personnel. The field of genomics is fast evolving, and integrating genomics courses and bioinformatics at the undergraduate level will build the awareness and competence as the younger generation scientists and physicians are well versed in genomics and do not shy away from embracing newer disciplines. There are large efforts underway internationally in universities and affiliated teaching hospitals to create precision medicine or precision oncology disciplines, and it should not be long before the new generation of biomarkers finds its place in health care.
